# Spleen Transcriptome Profiling Reveals Divergent Immune Responses to LPS and Poly (I:C) Challenge in the Yellow Drum (*Nibea albiflora*)

**DOI:** 10.3390/ijms24097735

**Published:** 2023-04-23

**Authors:** Xiang Zhao, Yuan Zhang, Tianxiang Gao, Na Song

**Affiliations:** 1 The Key Laboratory of Mariculture, Ocean University of China, Ministry of Education, Qingdao 266003, China; 2 CAS Key Laboratory of Tropical Marine Bio-Resources and Ecology, South China Sea Institute of Oceanology Chinese Academy of Sciences, Guangzhou 510301, China; 3 Fishery College, Zhejiang Ocean University, Zhoushan 316022, China

**Keywords:** *Nibea albiflora*, LPS, poly (I:C), transcriptome, functional genes, WGCNA

## Abstract

The yellow drum (*Nibea albiflora*) is a marine teleost fish with strong disease resistance, yet the understanding of its immune response and key functional genes is fragmented. Here, RNA-Seq was used to investigate the regulation pathways and genes involved in the immune response to infection with lipopolysaccharide (LPS) and polyinosinic-polycytidylic acid (poly (I:C)) on the spleen of the yellow drum. There were fewer differentially expressed genes (DEGs) in the LPS-infected treatment group at either 6 or 48 h. Kyoto Encyclopedia of Genes and Genomes (KEGG) analysis showed that these DEGs were mainly significantly enriched in c5-branching dibasic acid metabolic and complement and coagulation cascades pathways. The yellow drum responded more strongly to poly (I:C) infection, with 185 and 521 DEGs obtained under 6 and 48 h treatments, respectively. These DEGs were significantly enriched in the Toll-like receptor signaling pathway, RIG-I-like receptor signaling pathway, Jak-STAT signaling pathway, NOD-like signaling pathway, and cytokine–cytokine receptor interaction. The key functional genes in these pathways played important roles in the immune response and maintenance of immune system homeostasis in the yellow drum. Weighted gene co-expression network analysis (WGCNA) revealed several important hub genes. Although the functions of some genes have not been confirmed, our study still provides significant information for further investigation of the immune system of the yellow drum.

## 1. Introduction

The yellow drum (*Nibea albiflora*) belongs to the Sciaenidae family of the Perciformes order, being a eurythermic fish naturally distributed in the coastal waters of China, Japan, and Republic of Korea, with obvious seasonal migration habits [1]. In recent years, the yellow drum has become one of the most important commercially farmed fish in China due to its delicious taste, rich nutritional value, and the medicinal value of its otoliths and swim bladders [2]. With the rapid development of high-density net tank culture technology for the yellow drum, disease prevention and control during culture as well as cross-infection of different fish species cannot be ignored. In recent years, epidemiological investigations on marine fish have been carried out. Interestingly, the yellow drum was less susceptible to certain bacterial infections, more tolerant to white spot disease, and had a lower mortality rate than other farmed fish [3]. The immunomodulatory regulation mechanism of the yellow drum has attracted widespread attention, and related research has been carried out. Transcriptomic data from gill tissues of the yellow drum showed that toll-like receptors (*TLR5*) was the only receptor gene that activated the downstream immune response in early *Cryptocaryon irritans* infection (24 h). After 72 h, the expression of immunoglobulin-T-related genes was elevated [4]. In addition, the evidence from skin proteomics after stimulation of *Cryptocaryon irritans* infection demonstrated that 18 proteins associated with immunity and signal transduction in the yellow drum, such as malate dehydrogenase 2 (MDH2), malate dehydrogenase 1ab (MDH1ab), citrate synthase (CS), and complement component 3 (C3), interacted with each other, possibly contributing to the resistance and recovery of the yellow drum from the pathogen [5]. These results suggest that a rapid mechanism of innate and adaptive immune regulation may exist in the yellow drum. However, the mechanisms of immune regulation in the yellow drum remain largely unexplored.

In aquaculture, the role of immunostimulants in improving the natural immunity of aquatic products and reducing the risk of morbidity has received increasing attention. Polyinosinic-polycytidylic acid (Poly (I:C)) is a synthetic double-stranded RNA (dsRNA) viral analog [6]. Some studies have shown that poly (I:C) can induce a wider range of immune responses in fish. For example, microRNA-489 is involved in the immune response of miiuy croaker (*Miichthys miiuy*) after poly (I:C) stimulation [7]. Poly (I:C) can induce antiviral immune responses in Japanese flounder (*Paralichthys olivaceus*) [8]. Therefore, poly (I:C) is usually used as a mimic to explore the immune response of fish to viral infections [9]. In addition, lipopolysaccharide (LPS), a major component of the cell wall of Gram-negative bacteria, is recognized by a variety of pattern recognition receptors, which in turn triggers an immune response [10]. In recent years, the effect of LPS on immune regulatory mechanisms in teleost fish has been gradually emphasized. Although the results are sometimes not consistent, there is now growing evidence that LPS can induce an inflammatory response in teleost fish [11,12]. Therefore, LPS is usually used as a mimic to explore the immune response of fish to Gram-negative bacteria infections [13].

The aim of this study was, therefore, to explore the regulatory pathways and key genes in the response of the yellow drum to immunostimulants. Here, we designed an experiment with an intraperitoneal injection of poly (I:C) and LPS to examine the gene expression pattern of yellow drum spleen tissue at 6 and 48 h. The factors we expected to elucidate were as follows: (1) to reveal key regulatory pathways in yellow drum in response to viral and Gram-negative bacterial infections, (2) to identify key genes in the immune regulatory mechanism of the yellow drum, and (3) to provide a basis for an in-depth study of the immune system in the yellow drum.

## 2. Results

### 2.1. Data Filtering and Transcriptome Assembly

After filtering the raw data obtained from Illumina sequencing, about 868 million high-quality clean reads were produced. Both Q20 and Q30 of the data were above 94%, demonstrating the reliability of the sequencing data. All high-quality clean reads were mapped to the *Nibea albiflora* genome (GenBank: GCA_900327885.1), the mapping rate of the clean reads of each sample was higher than 90%, and a total of 21,615 genes were assembled.

### 2.2. Gene Expression Quantification and Differentially Expressed Genes (DEGs) Screening

**Figure 1:**
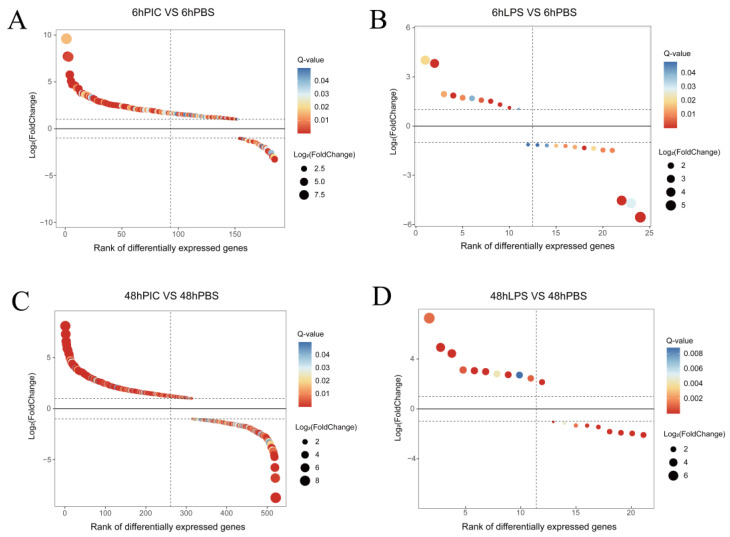
Sorting diagram of DEGs. (**A**) Overview of differentially expressed genes (DEGs) in 6hPIC vs. 6hPBS treatment. (**B**) Overview of DEGs in 6hLPS vs. 6hPBS treatment. (**C**) Overview of DEGs in 48hPIC vs. 6hPBS treatment. (**D**) Overview of DEGs in 48hLPS vs. 6hPBS treatment. Each dot in the figure represents a gene; above the dashed line are genes with upregulated expression, and below are genes with downregulated expression. The size of the dots represents the degree of differential expression—the larger the dot, the higher the degree of differential expression. The color indicates the significance test result—the closer to red, the smaller the Q-value, and the more significant the result.

To explore the gene expression pattern of the yellow drum under the poly (I:C) and LPS stress, we compared the number of DEGs in four treatment groups. In the 6 h attack experiment, 185 (153 up- and 32 downregulated) and 24 (11 up- and 13 downregulated) DEGs were obtained in 6hPIC vs. 6hPBS and 6hLPS vs. 6hPBS, respectively. In the 48 h attack experiment, 521 (314 up- and 207 downregulated) and 20 (11 up- and 9 downregulated) DEGs were obtained in 48hPIC vs. 48hPBS and 48hLPS vs. 48hPBS, respectively (Figure 1).

### 2.3. Gene Ontology (GO) Annotation and Kyoto Encyclopedia of Genes and Genomes (KEGG) Enrichment Analysis of DEGs

**Figure 2:**
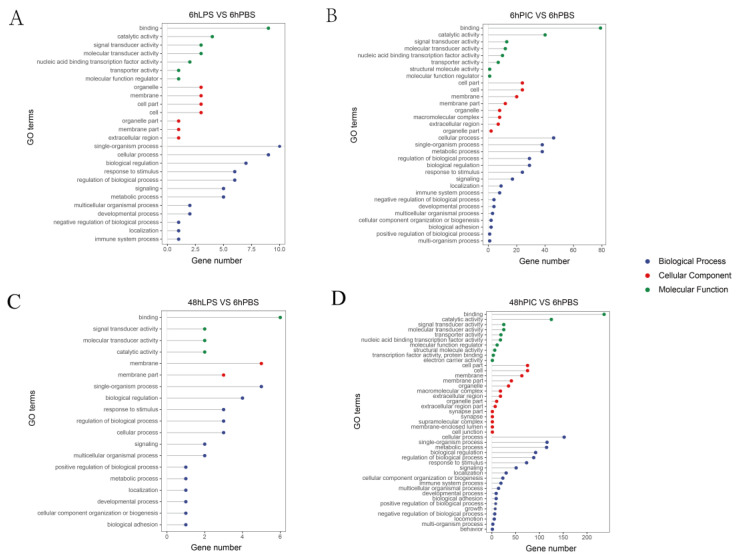
GO enrichment results of DEGs in the spleen of the yellow drum under lipopolysaccharide (LPS) and Polyinosinic-polycytidylic acid (Poly (I:C)) injection stress for 6 h (**A**,**B**) and 48 h (**C**,**D**).

To explore the immunomodulatory mechanism of the yellow drum, we carried out GO annotation and KEGG enrichment analysis of the DEGs of four treatment groups. The results showed that the main GO terms for DEGs included the cellular process (GO: 0009987), immune system process (GO: 0002376) of biological processes, binding (GO: 0005488) and catalytic activity (GO: 0003824) of molecular function, cell (GO: 0071944), cell parts (GO: 0044464), and membrane (GO: 0016020) of the cellular component (Figure 2).

**Figure 3:**
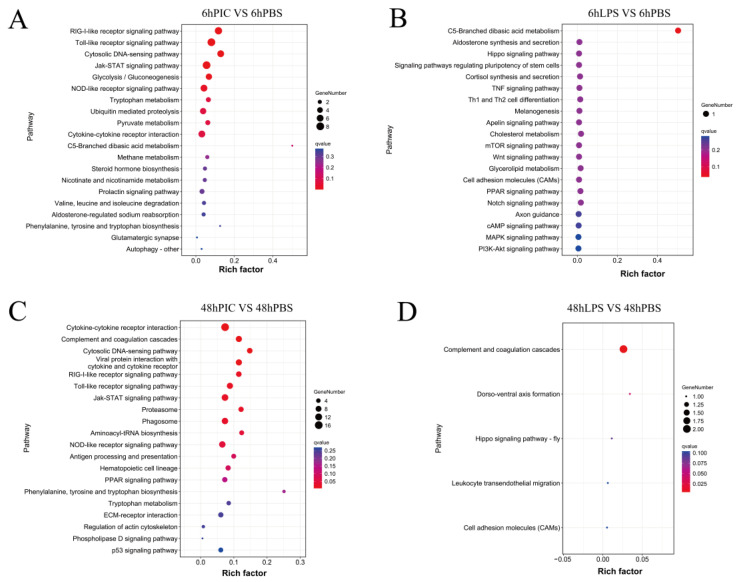
KEGG enrichment results of DEGs in the spleen of the yellow drum under LPS and poly (I:C) injection stress for 6 h (**A**,**B**) and 48 h (**C**,**D**).

**Figure 4:**
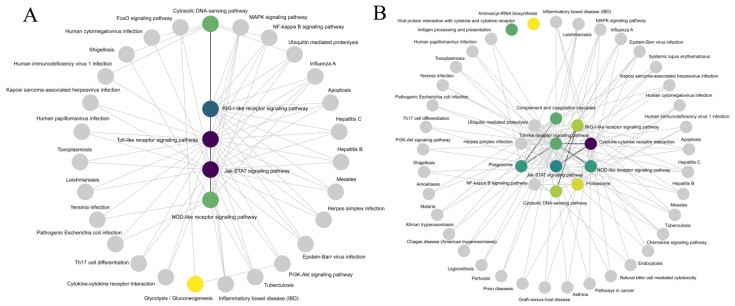
Interaction network of the KEGG pathway in poly (I:C) injection stress for the 6 h (**A**) and 48 h (**B**) treatment groups.

The enrichment analysis of the KEGG pathway provided valuable information for the study of the immunomodulatory mechanism of the yellow drum. At 6hLPS, we found that DEGs were only significantly enriched in the C5-branched dibasic acid metabolism (ko00660, Q = 0.039). However, DEGs were significant enriched in the RIG-I-like receptor signaling pathway (ko04622, Q = 0.000), Toll-like receptor signaling pathway (ko04620, Q = 0.000), cytosolic DNA-sensing pathway (ko04623, Q = 0.000), Jak-STAT signaling pathway (ko04630, Q = 0.000), glycolysis/gluconeogenesis (ko00010, Q = 0.007), and NOD-like receptor signaling pathway (ko04621, Q = 0.022) at 6hPIC. In the 48 h stress experiment, the DEGs were significantly enriched in the complement and coagulation cascades (ko04610, Q = 0.005) and dorso-ventral axis formation (ko04302, Q = 0.043) at 48hLPS. Interestingly, the DEGs were significantly enriched in the cytokine–cytokine receptor interaction (ko04060, Q = 0.005), complement and coagulation cascades (ko04610, Q = 0.005), cytosolic-DNA-sensing pathway (ko04623, Q = 0.005), viral protein interaction with cytokine and cytokine receptor (ko04061, Q = 0.005), RIG-I-like receptor signaling pathway (ko04622, Q = 0.019), Toll-like receptor signaling pathway (ko04620, Q = 0.020), Jak-STAT signaling pathway (ko04630, Q = 0.022), proteasome (ko03050, Q = 0.022), phagosome (ko04145, Q = 0.027), aminoacyl-tRNA biosynthesis (ko00970, Q = 0.042), and NOD-like receptor signaling pathway (ko04621, Q = 0.048) at 48hPIC (Figure 3). The KEGG pathway network showed an interaction between significantly enriched pathways (Figure 4).

### 2.4. Co-Expression Network Construction and Key Gene Screening Using WGCNA

**Figure 5:**
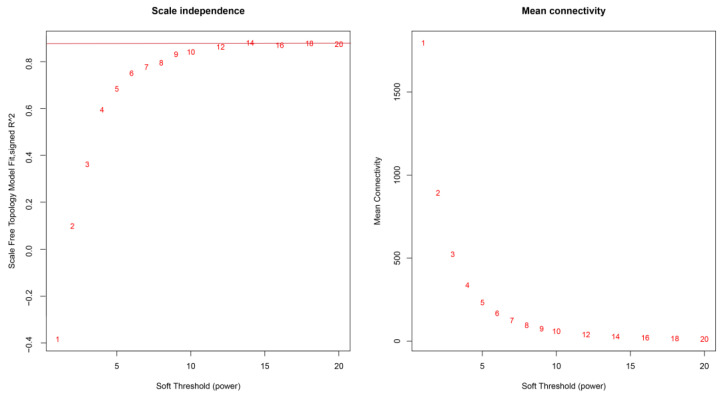
Determination of soft-threshold power in the WGCNA. The numbers 1–20 in the figure represent soft thresholds.

**Figure 6:**
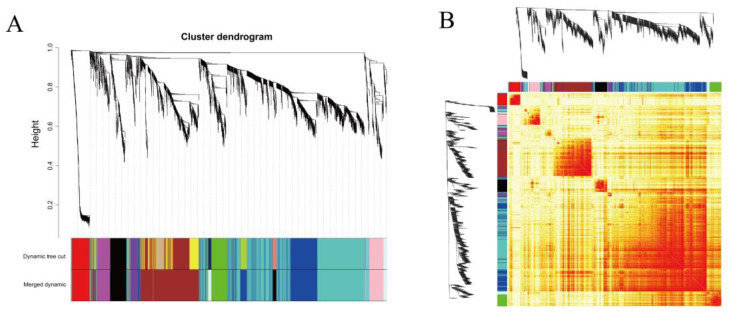
(**A**) Gene modules identified by weighted gene co-expression network analysis (WGCNA). This figure is divided into three parts: (1) gene dendrogram based on the TOM matrix; (2) gene module partition based on the dynamic programming algorithm; (3) final module partition result after optimization and merger. Each colored row represents a color-coded module that contains a group of highly connected genes. A total of 11 modules were identified. (**B**) Heat map of gene co-expression network with 1500 randomly selected genes.

**Figure 7:**
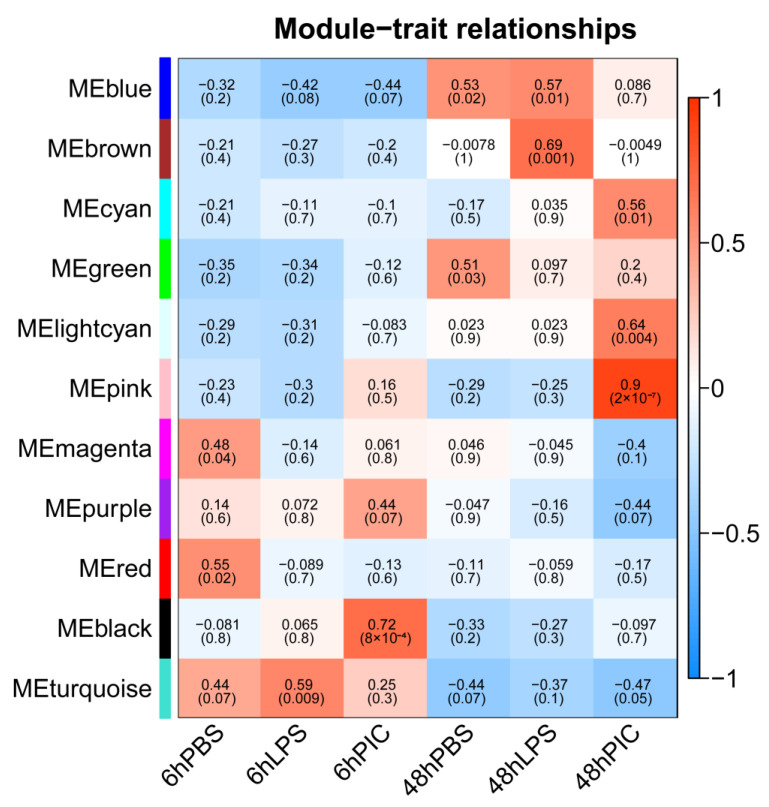
Heat map of correlation between co-expression modules and each treatment group.

**Figure 8:**
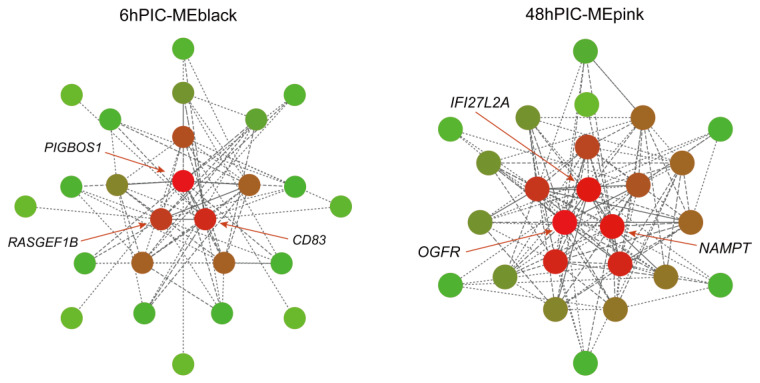
Gene co-expression network in the black and pink modules.

The sft$powerEstimate function was used to determine the soft power threshold β. The gene co-expression network conformed to the scale-free network distribution when β = 14 (Figure 5). Gene modules were then detected using the TOM matrix. Eleven modules were detected in the analysis (Figure 6). The correlation heat map showed significant correlation modules with some traits (Figure 7). The module significantly associated with 6hPIC was the black module, wherein the key hub-genes were the 54-amino-acid microprotein (*PIGBOS1*), *CD83*, and Ras-GEF domain-containing family member 1B (*RASGEF1B*). The module significantly associated with 48hPIC was the pink module, where the key hub genes were interferon-alpha-inducible protein 27-like protein 2A (*IFI27L2A*), opioid growth factor receptor (*OGFR*), and nicotinamide phosphoribosyl transferase (*NAMPT*) (Figure 8).

### 2.5. Validation of Transcriptomic Data via qRT-PCR

**Figure 9:**
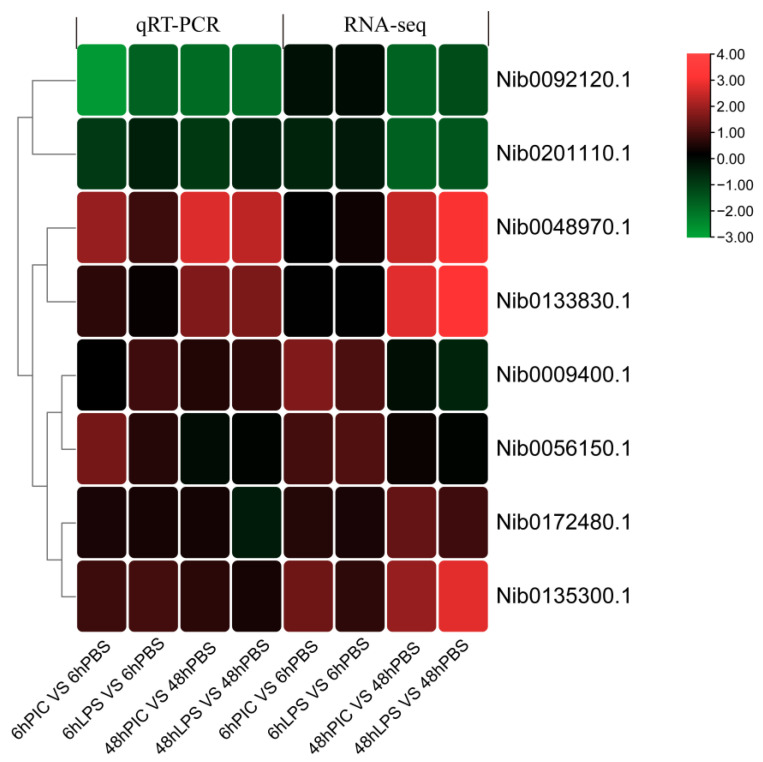
The relative change of the transcriptome data and qRT-PCR data of 8 DEGs.

Eight DEGs were selected on 18 cDNA templates for the qRT-PCR experiments. Melting curve analysis showed that a single product was amplified for all genes tested, indicating a largely accurate assembly of the transcriptome. The relative expression of the eight genes in the four treatment groups compared to the control group is shown in Figure 9. The results indicated that all genes tested conformed to the expression pattern of the transcriptome analysis, with only minor differences in expression levels. Overall, the results of qRT-PCR can confirm that the results of RNA-Seq expression analysis are reliable and accurate.

## 3. Discussion

Poly (I:C) and LPS were thought to activate an antiviral state in animals that last long enough to inhibit virus replication and protect fish from virus infection [14,15]. In this study, we used an intraperitoneal injection of poly (I:C) and LPS to simulate pathogen invasion and explored gene expression in spleen tissues of the yellow drum at 6 and 48 h on the basis of transcriptomic data. No mortality was observed in the yellow drum samples during the experiment. A total of 868 million high-quality clean reads were obtained in this study. Both Q20 and Q30 of the data were above 94%, demonstrating the reliability of the sequencing data.

### 3.1. Immunoregulatory Pathways and Key Genes under the LPS Injection Stress

Interestingly, there were very few DEGs in the LPS stress treatment group at either 6 or 48 h, and very few pathways were significantly enriched in the KEGG enrichment analysis. Previous studies have shown that the important immune response of the organism to LPS infection is the activation of the *TLR4* gene, which responds to LPS present in tissues and the bloodstream and triggers a pro-inflammatory response that promotes the eradication of invading bacteria [16]. The physiological effects of LPS may be mainly accomplished by activating the *TLR4* gene. However, our results showed that the *TLR4* gene was absent in the yellow drum genome (detailed analysis process and results are shown in the Supplementary Materials). Therefore, we hypothesized that the absence of the *TLR4* gene might inhibit the physiological effects of LPS. In addition, the yellow drum may have a high tolerance to LPS, and the low LPS concentration may be one of the reasons for the lower number of DEGs in the LPS-stressed treatment group. These speculations are reasonable, but further direct evidence is needed.

KEGG analysis showed that only the c5-branching dibasic acid metabolic pathway was significantly enriched in the 6hLPS group, and the clusterin gene (*CLU*) and tissue factor (*TF*) were significantly upregulated in this pathway. *CLU* was associated with apoptosis in zebrafish, and upregulation of this gene may protect the yellow drum cells from the cytolytic effects of complement [17]. Related studies have shown that *TF* is a transmembrane glycoprotein whose expression can be induced by a variety of stimuli, such as inflammatory cytokines, LPS, and growth factor [18]. The upregulation of *TF* in this study may be related to the repair of tissue damage and coagulation of the yellow drum under LPS stress. In addition, *TF* was able to indirectly mediate the signaling process through the activation of cell surface receptors via the coagulation cascade. In the 48hLPS, only the complement and coagulation cascades pathway was significantly enriched. Notably, *CLU* and *TF* were also significantly upregulated in the 48hLPS stress treatment group, and thus we speculated that these two genes may play an important role in the early and recovery process of the yellow drum in response to pathogen invasion.

### 3.2. Immunoregulatory Pathways and Key Genes under the Poly (I:C) Injection Stress

GO annotation results showed that many DEGs in 6hPIC and 48hPIC treatment groups were annotated to binding and cellular process terms. This is similar to the results of other studies on immune regulation in fish [19,20]. Multiple cellular interactions and signal transduction processes accompany the response mechanism of fish to viruses [21]. The interaction network of the KEGG pathway indicated that the Toll-like receptor signaling pathway, RIG-I-like receptor signaling pathway, Jak-STAT signaling pathway, NOD-like signaling pathway, and cytokine–cytokine receptor interaction were in the central regulatory position. Through in-depth exploration of genes in these pathways, we screened some functional genes that may be related to the immune regulatory mechanisms in the yellow drum. 

The innate immune system of teleost relies heavily on early responses to pathogens [22]. Chemokines are a superfamily of small molecule cytokines that elicit a chemotactic response or are cell-secreted signaling proteins with broad-spectrum effects on innate immunity [23]. In early immunity, chemokines can be used as initiators to recruit a variety of white blood cells to inflammatory sites [24]. C-X-C motif chemokine 10 (*CXCL10*) and C-X-C motif chemokine 13 (*CXCL13*) were significantly upregulated at 6hPIC and 48hPIC treatment, suggesting that poly (I:C) can induce a robust early immune response. Interleukins are a class of cytokines with multiple effects and critical immunomodulatory functions in both innate and adaptive immune responses [25]. Interleukin 6 (*IL6*) is a potent inducer of the acute phase response and contributes to host defense during viral infection and tissue injury [26]. Studies have shown that *IL6* plays an important role in the inflammatory response, lymphocyte differentiation, and antimicrobial peptide induction in teleost [27,28,29,30]. In addition, interleukin-10 (*IL10*) is thought to have an inhibitory effect on immune cell activation, inflammatory cytokine production, and resistance to bacterial pathogens [31]. Viral invasion of fish causes a strong inflammatory response, resulting in tissue damage. In our study, *IL6* and *IL10* were markedly upregulated in the 6hPIC and 48hPIC. We hypothesized that *IL6* first senses pathogen invasion signals and triggers a cascade of responses. *IL10* may attenuate virus-induced inflammatory damage by decreasing the expression of inflammatory factors. We also found that TNF receptor-associated factor 6 (*TRAF6*) was responsive to 6 h poly (I:C) stimulation. *TRAF6* is a crucial ubiquitin ligase in the Toll-like receptor signaling pathway. Related studies indicated that *TRAF6* can regulate the immune response of *Lateolabrax maculatus* by activating its NF-κB signaling pathway [32]. In zebrafish studies, *TRAF6* is able to induce serine/threonine-protein kinase *TBK1* to regulate inflammatory responses triggered by foreign factors [33]. Interestingly, *TBK1* expression was significantly upregulated under poly (I:C) stimulation in our results. We proposed that *TRAF6* senses viral signals at the early stage of poly (I:C) infection and activates downstream molecules of the antiviral innate immune process in the yellow drum. Similar to other related studies, we found that three interferon regulatory factors (*IRF2*, *IRF3*, and *IRF7*) were responsive to poly (I:C) stimulation [34,35]. IRFs are a family of transcription factors that play an essential role in the regulation of interferon (*IFN*) expression [36]. *IFN* can induce cellular resistance to viral infection by interfering with viral gene transcription or translation of viral protein components, thereby preventing or limiting viral infection [37]. Both *IRF2* and *IRF7* can bind directly to the IFNα/β promoter and positively regulate type I IFN production. In this study, *IRF2* was induced in the early phase of the antiviral response, while *IRF7* was induced both in the 6hPIC and 48hPIC. In addition, *IRF7* showed a larger upregulation fold compared to *IRF2*. These results suggested that *IRF2* and *IRF7* may play different roles in the activation of type I IFN in the yellow drum, but the mechanisms remain to be further investigated. In miiuy croaker (*Miichthys miiuy*) studies, *IRF3* and *IRF8* regulated the NF-κB signaling pathway by targeting MyD88. In the early stages of pathogen infection, the miiuy croaker (*Miichthys miiuy*) *IRF3* gene was significantly upregulated in expression to activate the NF-κB signaling pathway and trigger an immune response, whereas at 48 h of infection, *IRF3* was downregulated in expression and *IRF8* was upregulated in expression to maintain homeostasis of the immune system [38]. However, in this study, the expression of the *IRF3* gene was significantly upregulated at all time points, while *IRF8* did not change significantly. We proposed that *IRF3* may play an important role in both triggering the immune response and maintaining immune system homeostasis. Toll-like receptors (TLRs) in teleost fish are important for pathogen defense mechanisms in their innate immune system. In this study, *TLR9* was induced both in the 6hPIC and 48hPIC, and the *TLR9* gene has also been shown to activate the MyD88-dependent signaling pathway through the TIR functional domain, which is important for the regulation of innate immunity in the yellow drum [39], but the specific biological functions and host defense mechanisms of *TLR9* need to be further explored. In addition, *TLR3* and *TLR5S* were significantly upregulated only in the 48hPIC treatment group, suggesting that these two genes may not be involved in early pathogenic microbial recognition.

### 3.3. Key Hub Genes Related to Poly (I:C) Injection Stress Identified by WGCNA

WGCNA analysis showed that *PIGBOS1*, *CD83*, and *RASGEF1B* were the hub genes in the 6hPIC treatment group. PIGBOS1 is a microprotein located in the outer mitochondrial membrane, and related studies have shown that it can bind to the endoplasmic reticulum protein CLCC1 to stimulate unfolded protein responses [40]. Although *PIGBOS1* has not been studied in fish, the results of this study suggested that it may maintain cellular homeostasis in the yellow drum during the response to bacterial infection. *CD83*, a cell surface membrane glycoprotein, is a member of the Ig superfamily, and its expression can be significantly upregulated in immune organs such as the liver, thymus, and head kidney of sea bass (*Dicentrarchus labrax*) after poly (I:C) stimulation [41,42]. In mammals, *RASGEF1B* is a guanine nucleotide exchange factor mostly induced by *TLR3* and *TLR4* through the MyD88-independent pathway [43]. However, *TLR4* was absent and *TLR3* did not fold significantly under early poly (I:C) infection in the yellow drum, and thus we concluded that *RASGEF1B* in fish was not induced by TLRs. On the basis of the results of previous studies and the data of this study, we hypothesized that fish *RASGEF1B* may be induced by TGF-β signaling and thus exert immunomodulatory effects [44]. *IFI27L2A*, *OGFR*, and *NAMPT* were the hub genes in the 48hPIC treatment group. In mammals, *IFI27L2A* has been proven to play a role in the vascular injury response by regulating the transcriptional activity of *NR4A1* [45]. Although it has not been characterized in fish, we suggest that it may be a central regulatory gene in the repair of tissue injury and inflammatory response in fish. For Atlantic salmon, *OGFR* might be involved in the depletion of mucous cells infection of *Gyrodactylus salaris* (Monogenea) via suppression of DNA synthesis and a profound decrease in basal cell proliferation [46]. In this study, *OGFR* was significantly upregulated by 48 h poly (I:C) stimulation, and its connection value was high in the co-expression module, suggesting its importance in the immunity regulation in the yellow drum. In addition, *NAMPT* has been shown to induce the expression of antimicrobial molecules in hybrid Crucian carp (*Carassius auratus*), thereby enhancing host resistance to pathogens [47]. The results of this study also suggested its potential role in the yellow drum immune defense.

## 4. Materials and Methods

### 4.1. Ethics Statement

*Nibea albiflora* is not an endangered species. We anesthetized it with eugenol in field experiments to minimize the pain of all samples. All animal experiments were conducted following the guidelines and approval of the respective Animal Research and Ethics Committees of the Ocean University of China.

### 4.2. Experimental Fishes and Injection Program

The experimental fishes (10.1 ± 1.8 g) were from Aoshanwei National Marine Research Center, Qingdao City, Shandong Province, China. The fish samples used in this study have been cultured on the farm by using natural seawater (temperature: 20 °C; salinity: 31 psu; pH: 7.5) since they were bred. Poly (I:C) and LPS were dissolved in phosphate-buffered saline (PBS) and stored at −20 °C before the experiment. There is currently no uniform standard for LPS and poly (I:C) injection concentration, and this is because the species and size of the experimental fish will affect their tolerance to pathogens. For practical production, we hope to use LPS and poly (I:C) as an immunostimulant to activate the immune system of fish, instead of exposing fish to a lethal or semi-lethal state. Referring to several published studies, we found that injection concentrations of 1 µg to 50 µg LPS and poly (I:C) per gram of fish body weight were sufficient to trigger an immune response in fish. We chose a moderate concentration by considering the weight and tolerance of the fish in this experiment [48,49,50]. Referring to the sampling time of related experiments, the samples were collected at 6 h and 48 h after the injection experiment to investigate the immune response of the yellow drum at the early and later periods of pathogen infection [48,49,50]. Therefore, the experiment was divided into two parts: (1) The 6 h attack experiment: the experimental fishes were placed in 3 separate aquariums (80 × 60 × 40 cm) and 9 fishes per aquarium. The fish in 3 aquariums were injected intraperitoneally with 50 μL 2 mg/mL poly (I:C), LPS, and PBS, separately. After 6 h, 3 experimental individuals with similar size, vigor, and health were immediately euthanized and sampled in each experimental group (6hPBS, 6hPIC, 6hLPS). Spleen tissues were collected and quickly frozen in liquid nitrogen for RNA extraction. 6hPBS was used as the control group, and 6hPIC and 6hLPS were used as treatment groups. (2) The 48 h attack experiment: The experimental design was the same as the 6 h attack experiment, but the sampling time was 48 h after the attack. The 3 experimental groups were 48hPBS, 48hPIC, and 48hLPS. Among them, 48hPBS was used as the control group, and the 48hPIC and 48hLPS were used as the treatment group. A total of 18 spleen samples were collected in this study. The four groups of experiments of 6hPIC vs. 6hPBS, 6hLPS vs. 6hPBS, 48hPIC vs. 48hPBS, and 48hLPS vs. 48hPBS were analyzed.

### 4.3. RNA Extraction and Illumina Sequencing

In this study, the total RNA of the samples was extracted using a standard Trizol Reagent Kit following the manufacturer’s protocol. Then, the extracted total RNA was diluted according to a certain proportion before the concentration and integrity test. RNA concentration was measured using NanoDrop 2000 (Thermo Scientific, Waltham, MA, USA). RNA integrity was assessed using the Agilent RNA 6000 Nano Kit of the Agilent Bioanalyzer 2100 system (Agilent Technologies, Santa Clara, CA, USA). Only high-quality samples (OD260/280 ≥ 1.8, OD260/230 ≥ 1.8) were used to build a library for sequencing.

The mRNA with poly-A tail enriched by magnetic beads with Oligo dT was used to fragment RNA by interrupting buffer. Random N6 primers were reverse transcribed, and then double-stranded cDNA was synthesized to form double-stranded DNA. The ends of the synthesized double-stranded DNA were flattened and phosphorylated at the 5′ end, forming a sticky end protruding an “A” at the 3′ end, and then connecting a bubble-like joint with a protruding “T” at the 3′ end. The ligated products were amplified by PCR with specific primers. The PCR product was thermally denatured into a single strand, and then the single-strand DNA was cyclized with a segment of bridge primers to obtain a single-strand cyclic DNA library. Then, the sequencing was carried out by GooalGene Technology Company (Wuhan, China) on the Illumina Hiseq Nova platform. The raw data files were passed to NCBI’s Sequence Read Archive (SRA) (Bioproject ID: PRJNA846863). 

### 4.4. Data Filtering and Mapping

Raw sequencing data contains reads with low quality, compound contamination, and high unknown N. We filtered these reads using FASTP software (v0.23.1, Shifu Chen, Shenzhen, China) before subsequent analysis to ensure the reliability of the results [51]. The filtered “clean reads” were saved in FASTQ format. We then used HISAT2 software (v2.2.1.0, Daehwan Kim, Dallas, TX, USA) [52] to map the clean reads to the reference genome sequence (GenBank: GCA_900327885.1).

### 4.5. Differential Expression Genes (DEGs) Analysis and Functional Enrichment

To investigate the immunomodulatory mechanism of the yellow drum, we analyzed the number and biological functions of DEGs in the four treatment groups. First, we mapped all the clean reads onto the reference gene sequence using Bowtie2 [53]. Then, the gene expression levels of each sample were calculated to determine the fragments per kilobase of the exon model per million mapped fragments (FPKM) using RSEM with default settings [54]. According to the gene expression level of each sample, DEseq2 software (v1.38.3, Michael Love, Boston, MA, USA) was used to detect significant DEGs, and the |log2FC| ≥ 1 and Q value (adjusted *p*-value) ≤ 0.05 were used as the filtering thresholds [55]. We classified the DEGs of each treatment group by GO terms and the KEGG pathways. At the same time, we use the phyper function in R software (v4.1.0, Robert Gentleman and Ross Ihaka, Auckland, New Zealand) for enrichment analysis, and the GO terms and KEGG pathways of Q value ≤ 0.05 were regarded as significant enrichment. 

### 4.6. Weighted Gene Co-Expression Network Analysis (WGCNA)

WGCNA is a freely accessible R package for the construction of weighted gene co-expression networks [56]. We used the expression matrix of all genes in 18 samples for WGCNA analysis. The Pearson correlation coefficient between genes was used to construct a co-expression correlation matrix. Then, the weighted adjacency matrix was created using the formula a_ij_ = |s_ij_|^β^, where a_ij_ is the adjacency between gene i and gene j, s_ij_ is the Pearson correlation coefficient, and β is the soft-power threshold. Furthermore, the weighted adjacency matrix was transformed into a topological overlap measure (TOM) matrix to estimate its network connectivity property. The dissimilarity coefficient was used as the basic element to construct a clustering dendrogram of the TOM matrix and to identify the gene modules. The modules significantly associated with the high-temperature and low-temperature traits (Cor > 0.7, *p* < 0.01) were used for the follow-up analysis. The eigengene connectivity value was used to screen the hub genes in the modules. The minimal gene module size was set to 30 to obtain the appropriate modules, and the threshold for merging similar modules was set to 0.5. 

### 4.7. Quantitative Real-Time PCR Validation

We selected 8 differentially expressed gene fragments for qRT-PCR analysis on the basis of differential gene analysis. The specific primers of genes are designed by Primer Premier 6 software (v6.24, Premier Biosoft International, Palo, CA, USA), and the information of genes and primers is shown in Table 1. In addition, β-actin (F: 5′- CCTCCCTGGAGAAGAGCTATGAG-3′; R: 5′- CGCACTTCATGATGCTGTTGTAG-3′) and GAPDH (F: 5′- ACTGTCACTCCTCCATCTT-3′; R: 5′- GGTTGCTGTATCCGAACT-3′) were selected as reference genes for internal standardization. The total RNA used in the qRT-PCR experiment was the same as that used in Illumina sequencing. We used HiScript^®^ ⅡQ RT SuperMix for the qPCR kit (Vazyme, Nanjing, CHN) to reverse transcribe the total RNA of 15 samples to obtain the cDNA template used in the qRT-PCR experiment. According to the results of the standard curve, 18 cDNA samples were diluted 20-fold in nuclease-free water as templates for qRT-PCR. The QRT-PCR experiment was designed according to the instructions of SYBR^®^ PreMix Ex TaqTM (Tli RNase H Plus) RR420A and StepOnePlus (Takara, Tokyo, JPN). A 20 µL reaction system was amplified, including 10 µL of TB Green^®^ Premix Ex TaqTM (2×), 2 µL of cDNA template, 0.4 µL of forward and reverse primers and ROX Reference Dye (50×), and 6.8 µL of RNase-free water. The amplification processes consisted of a holding stage of 30 s at 95 °C, followed by 40 cycles of 5 s at 95 °C and 30 s at 60 °C. Three parallel experiments on each cDNA template were performed to reduce the error of the experimental results. The relative expression of each of the 8 genes was analyzed with the comparative cycle threshold (2^−ΔΔCT^) method (∆CT = CT_target gene_ − CT_reference gene_, ∆∆CT = ∆CT_treatment_ − ∆CT_control_).

## 5. Conclusions

In this study, there was a significant difference in the response to LPS and poly (I:C) infection in yellow drum. The minimal number of DEGs in the LPS injection group may have been due to the absence of *TLR4* in the yellow drum. C5-branching dibasic acid metabolic and complement and coagulation cascades are the key regulatory pathways in response to LPS infection. Poly (I:C) has excellent application potential in the immune enhancement of the yellow drum. The DEGs in the poly (I:C) injection group were significantly enriched in the Toll-like receptor signaling pathway, RIG-I-like receptor signaling pathway, Jak-STAT signaling pathway, NOD-like signaling pathway, and cytokine–cytokine receptor interaction. The key functional genes in these pathways played important roles in the immune response and maintenance of immune system homeostasis in the yellow drum. The results of the WGCNA analysis indicated that *PIGBOS1*, *CD83*, *RASGEF1B*, *IFI27L2A*, *OGFR*, and *NAMPT* may be pivotal genes in the immune response to viruses and host defense in the yellow drum. This study provided basic transcriptomic data for disease control and the development of targeted drugs in the yellow drum, as well as deepening the understanding of immune regulatory mechanisms in marine teleost fish.

## Figures and Tables

**Table 1:** Genes and gene-specific primers used for qRT-PCR.

Gene ID	Gene Name	Nr Annotation	Primer (5′ to 3′)
Nib0172480.1	*TLR22*	Toll-like receptor 22	F: TGAAGTGAAGGAGGACAATAC R: TGTAACTGATTCGGCAAGAT
Nib0092120.1	*TLR14*	Toll-like receptor 14	F: CAGGTGTTGGATGTTAGCA R: CTGATTGTGGTGAGCGATA
Nib0048970.1	*CLU*	Clusterin	F: TCAACACCTCCTCAATCCT R: TTCTCTTCTCCATCTGACTTC
Nib0133830.1	*WDFY1*	WD repeat and FYVE domain-containing protein 1	F: TGTTGCTGTATGCTGGAAG R: GTGTTGAAGACTTGACTGATG
Nib0135300.1	*CD36*	Coagulation factor IIIb	F: AGAACCTCTGGACTCTGATA R: ACGGCACTGATGTTACTC
Nib0201110.1	*GPCR68*	G-protein coupled receptor 68	F: GCAACTTCATCACAGGAATA R: CAACAGCAGCACAGAATC
Nib0009400.1	*NR4A1*	Nuclear receptor subfamily 4 group A member 1	F: AAGTGTTGGCAATCTGGAT R: GAGGCTGTGAGTAAGTTGT
Nib0056150.1	*NR4A3*	Nuclear receptor subfamily 4 group A member 3	F: CGACTACAGCCAGTTCAG R: GCAGAATCTATGAGCAGGTT

## Data Availability

The sequence data have been deposited in GenBank with no. PRJNA846863.

## Supplementary Materials

The following supporting information can be downloaded at https://www.mdpi.com/article/10.3390/ijms24097735/s1 [57,58,59].
