# Supplementary material for: Spleen Transcriptome Profiling Reveals Divergent Immune Responses to LPS and Poly (I:C) Challenge in the Yellow Drum (Nibea albiflora)
Source: Int J Mol Sci. 2023 Apr 23;24(9):7735. doi: 10.3390/ijms24097735 (PMC10178140; doi:10.3390/ijms24097735)
Supplement: Supplementary file 1 [file ijms-24-07735-s001.zip › ijms-2295161-supplementary.pdf]

We downloaded TLR protein sequences from NCBI database (https://www.ncbi.nlm.nih.gov/) for *Danio rerio*, *Larimichthys crocea*, *Cyprinus carpio*, *Miichthys miiuy* and *Argyrosomus japonicus* to construct blast database. BLASTP software (https://blast.ncbi.nlm.nih.gov/Blast.cgi) was used to perform homology searches for the yellow drum TLR protein sequences (threshold "--cut_tc" is higher than all false positive). Meanwhile, the HMMER search tool (Hidden Markov Model, HMM, version 3.2.0) was used to find the TLR domain (Pfam-B_571, http://pfam.xfam.org/) [57], and the yellow drum TLR protein sequences were searched globally by the HmmerSearch tool of the HMMER software [58]. Then both search results were intersected and short sequences less than 100 bp were excluded. A series of analysis tools used above were run on the Linux platform. Based on the nomenclature of the model species and closely related species, the yellow drum *TLR* gene family was named *NaTLRs*. the NJ phylogenetic tree was constructed using MegaX software, and the reliability of the phylogenetic tree was assessed using 1000 replicate sampling [59].

The results of sequence homology and evolutionary tree showed that the TLR gene family of the yellow drum includes *TLR1*, *TLR3*, *TLR5*, *TLR7* and *TLR11* subfamilies, with a total of 13 *TLR* genes and the absent of *TLR4* (**Figure S1**).

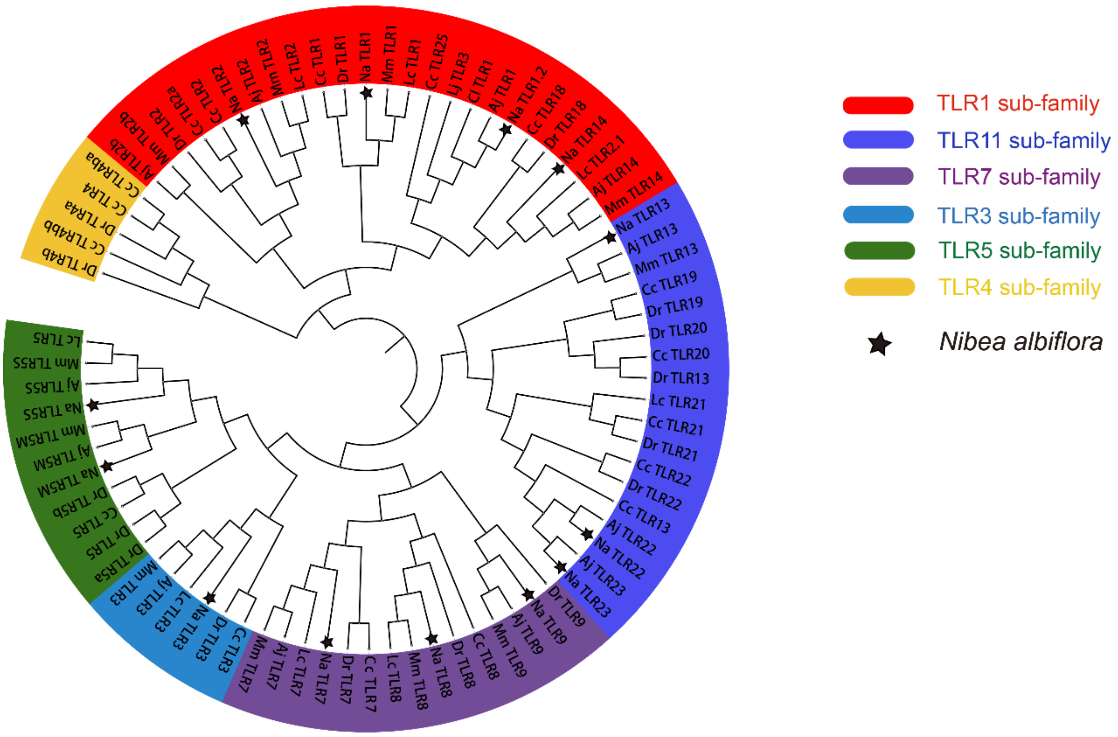

Figure S1. NJ phylogenetic tree based on TLR protein sequences of *Nibea albiflora* (Na), *Danio rerio* (Dr), *Larimichthys crocea* (Lc), *Cyprinus carpio* (Cc), *Miichthys miiuy* (Mm) and *Argyrosomus japonicus* (Aj).
